# Endocervix exhibits greater susceptibility to HIV-1 infection compared to ectocervix following *ex vivo* exposure to Transmitted/Founder HIV-1 variants

**DOI:** 10.1371/journal.pone.0334510

**Published:** 2025-11-05

**Authors:** Robert Langat, Michael D. McRaven, Ramon Lorenzo-Redondo, Teresia Muhomah, Ann M. Carias, Muhammad Shoaib Arif, Matrona Mbendo Akiso, Marianne Mureithi, Omu Anzala, Jill Gilmour, Sarah Joseph, Thomas J. Hope

**Affiliations:** 1 KAVI-Institute of Clinical Research, University of Nairobi, Nairobi, Kenya; 2 Department of Surgery, Division of Surgical Outcomes and Precision Medicine Research, University of Minnesota, Minneapolis, United States of America; 3 Department of Cell and Developmental Biology, Northwestern University, Chicago, Illinois, United States of America; 4 Department of Medicine, Division of Infectious Diseases, Feinberg School of Medicine, Northwestern University, Chicago, Illinois, United States of America; 5 Center for Pathogen Genomics and Microbial Evolution, Havey Institute for Global Health Northwestern University, Chicago, Illinois, United States of America; 6 Department of Medical Microbiology and Immunology, Faculty of Health Sciences, University of Nairobi, Nairobi, Kenya; 7 IAVI Human Immunology Lab, Imperial College London, London, United Kingdom; Icahn School of Medicine at Mount Sinai Department of Pharmacological Sciences, UNITED STATES OF AMERICA

## Abstract

The kinetics and identification of targets of Human Immunodeficiency Virus (HIV) infection within mucosae is a valuable tool for the development of new HIV-prevention strategies. Human tissue explants offer an informative model for studying HIV-1 pathogenesis and can support the development of novel HIV prevention interventions. Here, we infected cervical explants from HIV-1-uninfected women undergoing routine surgery with HIV_BaL_, a lab-adapted virus, and isolates HIV_4790_ and HIV_4791_, transmitted/founder (T/F) HIV-1 variants, and monitored the subsequent viral infection and replication using real-time quantitative PCR. The rates of infection and replication of HIV-1_BaL_ exceeded those of both HIV_4790_ and HIV_4791_. The two T/F isolates were not significantly different from each other overall in the explant comparison (endo and ecto cervical tissue combined); however, all three viruses demonstrated different tissue tropism. HIV-1_BaL_ and HIV_4790_ replicated at equivalent levels in endocervical explants, but HIV_4790_ replicated significantly less well in ectocervical explants. Alternatively, HIV_4791_ demonstrated inferior replication in endocervical tissues compared to HIV_BaL_ and HIV_4790_ but improved replication in ectocervical explants compared to HIV_4790_. Immunofluorescent analysis of the cervical tissues revealed the presence of viable immune cells that are targets of HIV-1 infection, thus validating our *ex vivo* model in its ability to maintain viable cells in culture for a longer period. This allows for assessing the dynamics of HIV replication in the cervical tissues. Our data suggests that endocervical tissues may be more susceptible to HIV-1 infections than ectocervix, revealing the complex dynamics across different sites of the lower female reproductive tract.

## Introduction

HIV and acquired immunodeficiency syndrome (AIDS) are global health challenges affecting millions of people across the world. As of 2023, it was estimated that 39.9 million people were living with HIV, with the majority of infections occurring in sub-Saharan Africa [1]. Among these individuals, women, particularly adolescent girls aged 15–19, are disproportionately affected, with four out of every five new infections occurring in this group. Furthermore, young women between the ages of 15 and 24 are twice as likely to be living with HIV as men of the same age, according to the same report. Unfortunately, although numerous HIV vaccine candidates have been developed and tested over 40 years, none have been effective. Only one clinical trial, RV144, has illustrated a modest benefit of reducing the risk of HIV infection with a vaccine efficacy of 31.2% [2], with other HIV therapeutic clinical trials showing, at best, no efficacy [3,4,5]. Therefore, HIV/AIDS is still here with us, and we urgently need an effective vaccine to curb or eradicate it. To achieve this, it is crucial to understand the earliest events that occur following sexual transmission at the female genital tract (FGT) mucosa, which serves as the initial portal of entry for the virus, before the virus eventually disseminates to the draining lymph nodes, establishing a systemic infection [6].

Intact mucosal surfaces, such as the FGT mucosa, provide a protective barrier against foreign pathogens; however, such pathogens can breach the epithelium to access the resident cells in the lamina propria [7,8]. For example, the FGT mucosa [6,7,8] is the initial site of replication for sexually transmitted infections, including herpes simplex virus type 2, human papillomavirus, and HIV [9,10]. The FGT is made up of two compartments: the stratified epithelium lining the ectocervix and vagina of the lower FGT and the simple columnar epithelium lining the uterus, fallopian tubes, and endocervical canal of the upper FGT. The susceptibility of these compartments to HIV-1 infection is not fully understood. Studies using the nonhuman primate and human *ex vivo* models have reported conflicting results, with some showing that the endocervix is highly susceptible to simian immunodeficiency virus (SIV)/HIV-1 infection relative to the ectocervix [11,12], while others reported the ectocervix as more susceptible to HIV-1 strains than the endocervix [13]. Additionally, Stieh et al. illustrated that the entire upper and lower FGT can be infected [14].

To investigate the susceptibility of endocervix and ectocervix to HIV-1 infection, we utilized an *ex vivo* culture model previously described with slight modifications where we challenged endocervical and ectocervical explants with HIV-1_BaL_ and HIV-1 T/F variants in a non-polarized way [15–18]. The culture of tissue explants at the liquid-air interface, using gelatin sponges as support, maximizes exposure to air oxygen while providing access to culture medium nutrients through the sponge capillaries, thus delaying their natural decay. We evaluated the HIV-1 infection and replication by measuring the HIV-1 DNA by real-time quantitative PCR. We also employed immunofluorescent staining to identify the lamina propria and epithelium and evaluate tissue integrity to ensure we had intact lamina propria and epithelium for analysis. Further, IF allows us to assess the immune cells within the mucosal tissue environment.

Here, we report the susceptibility of endocervical and ectocervical to HIV-1_BaL_ and HIV-1 T/F variants from the same participants, comparing the rates of HIV-1 infection and replication amongst the three viruses in the explants (endo and ecto cervical tissues). While we did not find significant differences in HIV-1 infection and replication between HIV-1_4790_ and HIV-1_4791_ overall (ecto and endo cervix), the three viruses demonstrated different tissue tropism with Bal and HIV_4790_ replicating at equivalent levels in endocervical explants, but HIV_4790_ replicated significantly less well in ectocervical explants. Our findings highlight additional information about HIV-1 T/F variants infectivity in the cervical mucosa that has not been previously reported, which may underlie the differential vulnerability of endo and ectocervical tissues. The *ex vivo* human cervical tissue model offers a unique opportunity to study the susceptibility of different parts of the cervix to HIV-1 infection. Here, we investigated the susceptibility of the endocervix and ectocervix to HIV T/F variants and lab-adapted reference strain HIV_BaL_ *ex vivo*. Others and our lab have shown that this model can maintain viable immune cells, such as T cells, B cells, and macrophages, as well as follicular dendritic cell networks in lymphoid and cervical tissue cultures. for two weeks [15,19,20–24].

## Materials and methods

### Participants, samples, and data collection

This study enrolled 59 women from two well-established cohorts at Kenyatta National Hospital in Nairobi and Northwestern Memorial Hospital in Chicago from October 6, 2017 to April 14, 2022. Cervical tissues from routine hysterectomies were obtained through protocols approved by the Kenyatta National Hospital – University of Nairobi Ethics and Research Committee (P60/02/2017, and P819/09/2019) and Northwestern University (STU0025456-CR0002 and STU00209475). Women undergoing routine hysterectomies were eligible if they were aged 18–60 years and willing to provide informed consent. Exclusion criteria included HIV infection, any condition that, in the investigator’s judgment, could compromise the participant’s health, hinder consent, or affect study outcomes. We also collected information on contraceptive use, menstrual history, last sexual intercourse encounter, use of condom, and HIV status. All recruited participants provided written informed consent before participating in the study.

### Preparation of viral stocks

The following viruses were obtained through the NIH HIV Reagent Program, Division of AIDS, NIAID, NIH: HIV-1 BaL [25]; plasmids encoding the infectious molecular clones, HIV-1_4790_ and HIV-1_4791_ were generously provided by Dr. Eric Hunter at Emory University. These clones were generated from samples drawn from the International AIDS Vaccine Initiative (IAVI) Protocol C early- HIV infection cohort [26] and generated at Emory University, Atlanta, USA, as previously described [27,28]. The plasmids were amplified in One Shot Stbl3 Chemically Competent E. coli (Thermo Fisher Scientific) and purified using the Endo Free Plasmid Maxi Kit (Qiagen, Valencia, CA). Sequence homology between original and amplified plasmids was confirmed by digestion with restriction enzymes and gel electrophoresis. 293T cells (Invitrogen) were transformed with plasmids using polyethyleneimine Reagent (Biosciences). Culture supernatants were harvested 42, 48, and 66 hours post-transfection, filtered, aliquoted, and stored at −80 °C. Cell-free viral stocks for the clade B reference strain HIV_BaL_ and clade C T/F HIV-1 variants, HIV-1_4790_, and HIV-1_4791,_ were produced in peripheral blood mononuclear cells isolated from multi-donor buffy coats from healthy HIV-1-seronegative donors as previously described [14]. Culture supernatants were harvested at day 11 post-infection, passed through a 0.45μm filter, ultracentrifuged at 100,000 rpm for 2 hours at 4°C, aliquoted, and stored at −80 °C.

### Viral stock Titration

The viruses were titrated using the tissue culture dose for 50% infectivity (TCID_50_) technique. Briefly, 4–5 × 10^7^ PBMCs were resuspended at a density of 2 × 10^6^ cells/mL in RPMI media supplemented with 10% FBS, IL-2 at 20 U/mL, PHA at 1 mg/mL, and Zosyn at 0.5 mg/ mL and cultured for 3 days at 37°C with 5% CO_2_. After washing the cells at 300 × g for 10 minutes, they were counted and resuspended to a final concentration of 6 × 10^6^ cells/mL. The viruses to be tested were serially diluted by adding 70 μL of the virus to 7 mL of the RPMI media above to make a starting dilution of 10^-2^, then 700 uL of this dilution was added to 6.3 mL media to make 10^-3^, 10^-4^, 10^-5^, etc., depending on the number of cell replicates available. A 1 mL cell suspension (6 × 10^6^ cells) was then pipetted into 15 mL conical tubes and spun down to remove the supernatant at 300 × g for 10 minutes. The supernatant was then removed, and the cell pellet was resuspended by gently adding 6 mL of the viral dilution to each tube. The cells and virus were cultured at 37°C with 5% CO_2_ for 4 hours, with gentle mixing every hour. After incubation and washing, the cells were resuspended in fresh RPMI media containing 10% IL-2 and 0.5 mg/mL Zosyn at a final cell density of 1 × 10^6^ cells and plated at 1 mL per well in a 48-well plate. The cells were cultured at 37°C with 5% CO_2_ with media changes every 3–4 days up to day 14. The supernatant was collected and frozen on days 7 and 14 for p24 ELISA. TCID_50_ was calculated from the p24 result using the Reed and Muench endpoint calculation method, as described previously [29]. Identical stocks were generated and used throughout the study to control for any inherent variations between batches and to ensure that the data collected is comparable.

### Sample preparation

Cervical tissues were processed within four hours of surgery, following a published protocol with slight modifications [19]. Briefly, the cervical tissue was dissected into the endocervix and the ectocervix based on anatomical structure. The mucosa of the endocervix and ectocervix was then separated and removed as much as possible, leaving a thin layer of tissue that contained the underlying mucosa and epithelium. The tissue was then cut into small strips approximately 2 mm in size. From these strips, we cut them further into small blocks about 2 mm thick. This resulted in blocks of approximately 8 mm³, which were kept submerged in a Petri dish containing culture media to prevent tissue desiccation until ready for infection. Three to five cervical explants were used for each experiment.

### Infection of ectocervical and endocervical explants with HIV-1 variants

Donor-matched endocervical and ectocervical explants were either infected with HIV_BaL_, HIV_4790_, or HIV_4791_ (at 1 × 10^4^ TCID_50_/mL) in a non-polarized way. Mock-infected and nevirapine (NVP) controls were included in all conditions, and the tissue preparation and experimental conditions remained identical throughout the study. Infection was done by submersion in an Eppendorf tube containing R10 media and viruses. Infected explants were incubated for 2 h at 37 °C, rocking at 300 revolutions per minute. To differentiate between the viral inoculum and the *de novo* production of HIV, endocervical and ectocervical explants were pretreated with NVP for 4 h before infection and subsequently added at every medium change until the end of the culture. After infection, the tissue blocks were extensively washed with 1X phosphate-buffered saline (PBS) and then transferred into pre-wet collagen sponges (Spongostan Standard 7 × 5 × 1 cm, Johnson & Johnson Medical NV, Diegem, Belgium). Five to 6 blocks were placed on each sponge in 6-well plates containing 4 mL of R10 media and cultured for 12 days at 37 °C. Cultures that showed clear signs of bacterial contamination were discarded.

### HIV-1 DNA quantification in cervical tissues

#### Extraction of total cellular DNA.

Cervical explants were subjected to collagenase digestion, mechanical disruption using a scalpel to cut them down into small pieces, clarification (by passing through a 70 µm cell strainer), and washing. Total cellular DNA was extracted from the clarified cells using 0.5 ml of back extraction buffer, followed by precipitation with 500 µl of isopropanol, 2 washes with 75% ethanol, and resuspension in 100 µl of EB buffer DNeasy Blood and Tissue kits (Qiagen, Valencia, California, USA. Isolated DNA concentrations were measured using Nanodrop (GE Healthcare Life Sciences, Piscataway, NJ) and frozen at −80 °C until use.

#### Quantitative PCR for viral DNA.

Two replicate samples of DNA from each donor were assayed for total HIV-1 DNA using a modification of a published real-time TaqMan PCR assay that uses primers and a probe from the LTR region [30]. Primers were F522-43 Kumar (*5′ GCC TCA ATA AAG CTT GCC TTG A 3′*; HXB2 522–543) and R626-43 Kumar long (*5′ GGG CGC CAC TGC TAG AGA 3′*; 626–643). The probe, *5′ CCA GAG TCA CAC AAC AGA CGG GCA CA 3′*, was dual-labeled with 6-FAM (5′) and Black Hole Quencher BHQ-1 (3′). The primers for GAPDH were a forward primer (5’-GAA GGT GAA GGT CGG AGT-3’) and a reverse primer (5’-GAA GAT GGT GAT GGG ATT TC-3’ [31]. The reaction volume was 25 µl with 12.5 µl of ABsolute Blue qPCR Low ROX Mix or ABsolute QPCR SYBR Green Low ROX Mix (Thermo Fisher Scientific) or 5 pmol of each primer, 5 pmol of the probe, and 5 µl of DNA. Cycling conditions were: 95 °C for 15 min, then 40 cycles of 95 °C for 15s, and 59°C for 1 min. External standards (10^6^ to 1) were prepared from DNA extracted from known numbers of 8E5 cells (NIH AIDS Reagent Program), each of which contains one integrated HIV-1 genome per cell. Glyceraldehyde 3-phosphate dehydrogenase (GAPDH) was used as a housekeeping gene to normalize HIV-1 DNA numbers per cell.

### Analysis of tissue samples by immunofluorescent microscopy

Frozen tissue explants in optimal cutting temperature compound were sectioned 12-µm-thick using a cryostat and placed onto frosted microscope slides. Tissue sections were fixed with PIPES (piperazine-N, N’-bis (2-ethane sulfonic acid)) buffer containing 3.7% formaldehyde and incubated for 10 min at room temperature (RT). Next, sections were washed with cold 1X PBS, blocked with normal donkey serum (Sigma-Aldrich, USA) for 10 min, and washed as before. To identify HIV-1 target cells, tissue sections were stained with anti-CD3 (clone SP7, Abcam), anti-CCR6 (clone G034E3, BioLegend), and anti-HIV-Gag-Ag3 hybridoma (NIH AIDS Reagent Program), each for 1 hour at 37°C. Next, tissue sections were stained with secondary antibodies conjugated with rhodamine RedX, Cyanine 5 (Cy5), or Alexa Fluor 488 (all Jackson Laboratories, USA), incubated for 30 min at RT, and washed as before. To stain for nuclei, slides were stained with DAPI (Invitrogen, Cat#D1306, Stockholm, Sweden) for 10 min at RT. Slides were thoroughly washed in PBS, dried, mounted with DakoCytomation and a cover slip, and allowed to set for 1 h. Four color (DAPI, AlexaFluor488, TRITC, AlexaFluor 647) image stacks containing 20–40 sections in the Z plane in 0.5 mm steps were acquired and deconvolved using softWoRx software (Applied Precision) on a DeltaVision inverted microscope.

### Statistical analysis

Statistical analysis and graphical presentations were performed using GraphPad Prism statistical software version 9 (GraphPad Software, San Diego, CA) and the R statistical version 3.5.0 (R Core Team, Vienna, Austria) computing environment. Results were analyzed by pairwise comparison with the Wilcoxon signed-rank test between different pairs of groups’ nonparametric data, and when three or more groups were compared, a linear mixed-effects model with false discovery rate (FDR)- adjusted p-values for multiple comparisons was performed. We evaluated significant differences in the frequency of infection by fitting a binomial generalized linear mixed-effects model to a binary outcome for infection where samples with more than 26 copies/mL of *Gag* (our qPCR limit of detection) were considered positive for an infection and below that, negative. In this model, we included virus, tissue type, and their interaction. We evaluated statistically significant differences in HIV replication by fitting a linear mixed-effects model to the HIV-1 DNA copies normalized by GAPDH per sample, assuming a log-normal distribution and including virus, tissue type, and their interaction. Pairwise comparisons between each group tested were performed. Nominal *p* values of <.05 were considered significant, but for each variable, we further computed FDR *q* values using the Benjamini-Hochberg method to accommodate multiple testing. Statistically significant differences and p-values were FDR-adjusted. The model accommodated the combination of data from more than one experimental condition to increase the power of analysis and allowed comparison of the T/F HIV-1 variants against HIV_BaL_ as a reference control. HIV-1 DNA copies are presented as median and interquartile ranges.

## Results

### Sociodemographic characteristics of participants

The sociodemographic characteristics of all the 59 participants included in the study are summarized in **Table 1**. The median age (interquartile range) was 45 (41–51) years, with the majority (81%) being ≥40 years. The main reason for hysterectomy was fibroids (56%). Several women were multiparous and were not using any contraceptives at enrollment. We obtained matched endocervix and ectocervix from most (85%) donors.

**Table 1:** Sociodemographic characteristics of study participants.

Variable	Frequency, *n *= 59
**Age, Median (IQR)**	45 (32–33)
**Race, No. (%)**	
Black	52 (43)
White, non-Hispanic or Latino	7 (12)
**Reason for surgery, No. (%)**	
Uterine fibroids	33 (44)
Endometrial hyperplasia	3 (5)
Abnormal uterine bleeding	9 (15)
Uterine prolapse	6 (10)
Ovarian mass	4 (7)
Uterine adenomyosis	4 (7)
**Tissue type, No. (%)**	
Endocervix	4 (7)
Ectocervix	5 (8)
Endocervix + ectocervix	50 (45)

### Endocervical and ectocervical explants are productively infected with T/F HIV-1 variants

**Fig 1:**
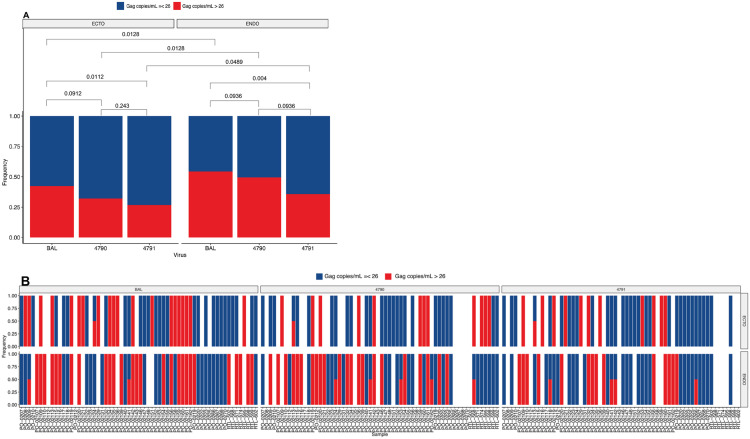
T/F HIV-1 variants (HIV_4790_ and HIV_4791_) and HIV_BaL_ established infection in human cervical tissues *ex-vivo.* Matched endocervical and ectocervical explants were infected with either HIV_BaL_, HIV_4790_, or HIV_4791_ and cultured for 12 days at 37°C. **A.**) Combined samples by tissue type and virus, and **B.**) per sample. Frequency of HIV-1 infection as determined by HIV-1 DNA copies/mL, with > 26 copies considered positive (productive infection) in red and <26 copies negative (no infection) in blue.

We investigated whether T/F HIV-1 variants (HIV_4790_ and HIV_4791_) could infect *ex vivo* human cervical tissues and compared their infectivity to that of HIV_BaL_. To determine this, the presence or absence of HIV-1 DNA in the explants was first measured, followed by quantification of HIV-1 DNA copies in productively infected cervical tissues. Our data revealed that most infections occurred in donor-matched endocervical and ectocervical tissues infected with HIV_BaL_, followed by HIV_4790_ and HIV_4791_, suggesting that HIV_BaL_ was more efficient at infecting cervical tissues (**Fig 1**). Specifically, HIV_4791_ showed significantly fewer infections in both endocervical and ectocervical tissues compared to HIV_BaL_ (p = 0.0112 and p = 0.004, respectively). Although lower than HIV_BaL_, HIV_4790_ infection was almost comparable in both endocervical and ectocervical (p = 0.0912 and p = 0.0936, respectively) (**Fig 1A**). There were no differences between HIV_4790_ and HIV_4791_ infection in either ectocervical tissues (p = 0.243) or endocervical tissues (p = 0.0936).

Furthermore, we observed that endocervical tissues were more susceptible to infections than donor-matched ectocervical tissues, regardless of the virus type (**Fig 1B**). Specifically, HIV_BaL_ infected more endocervical tissues than ectocervical tissues (p = 0.0128), and this trend was also observed for HIV_4790_ and HIV_4791_ (p = 0.0128 and p = 0.0489, respectively) (**Fig 1A**). These findings suggest that T/F HIV-1 variants and HIV_BaL_ can penetrate simple columnar epithelia and overcome the mucus barrier to access resident HIV-1 target cells in the submucosa, thereby establishing infection. Taken together, our data reveal that in cervical tissue explants, T/F HIV-1 variants can establish infection, with no significant differences observed in their replication patterns. These findings contribute to our understanding of the earliest HIV-1 transmission events and may inform prevention strategies.

### Ecto- and endocervical tissues supported HIV-1 replication in *ex vivo* culture

**Fig 2:**
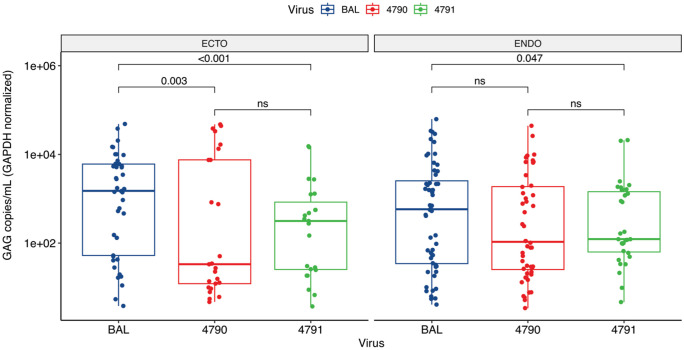
Donor-matched cervical tissues inoculated with HIV-1 became productively infected. Endocervical and ectocervical tissues were infected with either HIV_BaL_, HIV_4790_, or HIV_4791_ and cultured for 12 days. The boxplot shows the median, with quartile ranges and whiskers extending to 1.5 times the max and min-quartile values of the number of HIV-1 DNA copies on day 12. A single point represents each analyzed sample. Kruskal-Wallis test was conducted for each comparison shown, and the multi-comparisons analysis was corrected by FDR using the Benjamini-Hochberg method. The adjusted p-value is displayed when p ≤ 0.05, and the p-values are labeled as “ns” for not significant when the adjusted p > 0.05.

Following successful infection of the cervical tissues by the HIV-1 T/F variants and HIV_BaL_, we next investigated whether these tissues could sustain HIV-1 replication over time by measuring the HIV-1 DNA copies. We used GAPDH as a housekeeping gene to normalize HIV-1 DNA copies per cell to ensure we quantified equivalent genomic material across the tissues. There were no significant differences in GADPH copies between the tissues (S1 Fig). Our data reveal that endocervical tissues infected with T/F HIV-1 variants had higher HIV-1 DNA copies than the matched ectocervical tissues, in contrast with HIV_BaL,_ which had higher copies in ectocervical tissues than endocervical tissues (**Fig 2**). The HIV-1 DNA copies in HIV-1_BaL_ infected endocervical tissues were significantly lower than matched ectocervical tissues, with a median (IQR) of 3,709 (251−19,491) copies/mL compared to 11,438 copies/mL (334−43,879) in ectocervical tissues (p = 0.006; **Fig 2**). HIV_BaL_ was used as a reference point, and when we compared the replication of HIV_BaL_ versus T/F HIV-1 variants, the HIV DNA copies were significantly lower in tissues infected with HIV-1 T/F. For instance, HIV DNA copies in HIV_4791_ infected endocervical tissues were 943 (438−8,483) copies/mL, significantly lower than those observed in HIV_BaL_ endocervical tissues (p = 0.047). The same was seen in HIV_4791_ infected ectocervical tissues with 2303 (168−6,716) copies/mL, which was also significantly lower than HIV_BaL_ (p < 0.001; **Fig 2**). Interestingly when we compared HIV_BaL_ with HIV_4790_, we only observed differences in ectocervical tissues, where the HIV DNA copies in HIV_4790_ infected ectocervical tissues were significantly lower than HIV_BaL_ infected tissues with 259 (90–53,497) copies/mL (p = 0.003), but similar in endocervical tissues with 776 (187−11,004) copies/mL (p = 0.06; **Fig 2**, S1 Table). This suggests that HIV_4791_ produced less virus in ecto and endocervical tissues, while HIV_4790_ produced less virus only in ectocervical tissues. When compared, the copies of HIV_4790_ and HIV_4791_ were similar in both endocervical and ectocervical tissues (p = 0.701). These results show that T/F HIV-1 variants can replicate to high levels in an ex vivo culture model under controlled laboratory conditions, and cervical explants can maintain viable immune cells susceptible to HIV-1 in culture over time, underscoring their importance in HIV infection studies.

### T-lymphocytes were distributed throughout the tissue but primarily concentrated below the basement membrane

**Fig 3:**
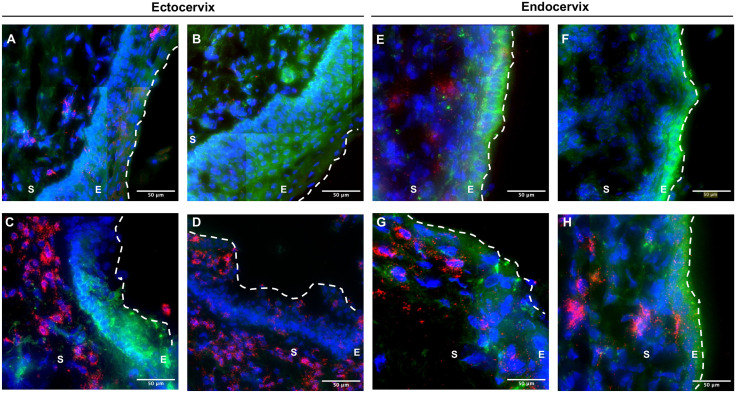
Immunofluorescent imaging of the cervical tissue and distribution of the CD3^+^ T cells. Representative immunofluorescent stained tissue section from ectocervix and endocervix images from 4 donors. DAPI stain is shown in blue, the CD3 stain is in red, and the background is in green. Dashed lines indicate the luminal surface—white dotted line, lumen; E, epithelium; S, stroma.

*Ex vivo* explant models have become a primary model for studying HIV pathogenesis, allowing for the visualization of immune cells within the tissue. To assess the localization and concentration of potential HIV-1 target cells important in HIV transmission, we performed immunofluorescence imaging on frozen sections infected with HIV-1. We imaged the tissues collected 48 hours and 12 days post-infection. Here, we focused on CD3^+^ T cells, as these are the primary targets of HIV-1 infection [12,32–38]. We purposefully stained for CD3^+^ T cells instead of CD4^+^ T cells as CD4 is internalized and degraded in HIV-1 infected cells, resulting in decreased fluorescent intensity to levels comparable to those seen in uninfected cells [14,39,40]. Also of importance was that we assessed the tissue’s structural stability during the infection process. In this *ex vivo* culture model, the tissue remained intact, as evidenced by a squamous epithelium with a visible luminal surface, lumen, and stroma in ectocervical explants, as shown in Figs 3A and B. We observed the distribution of CD3^+^ T cells throughout the tissue with a concentration below the basement membrane (Figs 3C and D). For the endocervical explants, the simple columnar epithelium remained intact (Figs 3E and F), and the CD3^+^ T cells were distributed throughout the epithelium and lamina propria (Figs 3G and H). This is consistent with previous studies that showed a concentration of CD3^+^ T cells below the basement membrane and within the squamous epithelium of the ectocervix and ectocervix [16,41]. While our primary focus was not on determining the differences in CD3^+^ T cell numbers between endocervix and ectocervix, but instead on assessing the stability of the tissues in culture over time, these observations could potentially correlate with the abundance and localization of immune cells within the tissue, with the differential susceptibility of endocervical and ectocervical tissues.

### Diverse immune cell phenotypes are susceptible to HIV-1 infection

**Fig 4:**
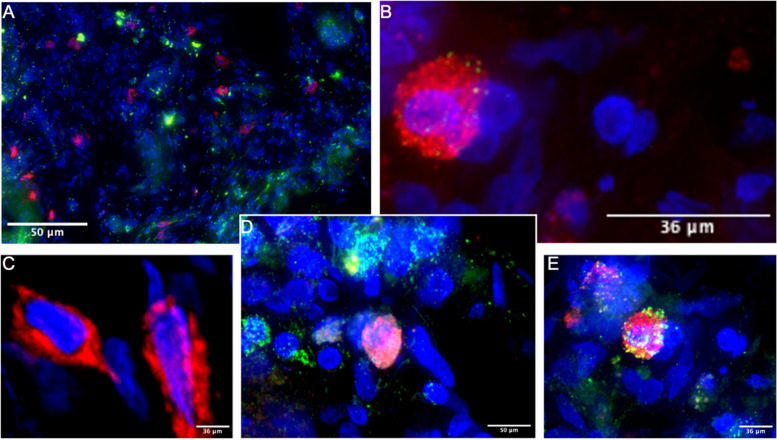
Diverse cell phenotypes were susceptible to HIV-1 infection. **(A-C)** Representative immunofluorescent stained tissue section from ectocervix. The DAPI stain is blue, the Gag stain is red, the CD3 stain is green, and the CCR6 stain is green. **A)**The infected cells are distributed throughout the stroma. **B-E)** Zoomed images of the infected cells: **B-C)** CD3^-^CCR6^-^, **D)** CD3^+^ T cells colocalized with infected cells, and **E)** CD3^+^CCR6^+^ infected cells.

It has been reported that diverse immune cells are susceptible to HIV-1 infection in the FRT [42–44]. We performed immunofluorescent staining on endocervical and ectocervical tissue sections to determine the phenotype of infected cells in the cervical tissues. We focused on characterizing diverse T cell markers, as CD3^+^ T cells constitute the predominant lymphocyte population in the lower FRT, and these cells are primary targets of SIV/HIV infection [14,42]. However, not all subsets of CD3^+^ T cells are equally susceptible to infection, and different subsets of T cells are depleted at varying rates from the mucosa [14,43–48]. Th17 cells, a major CD4^+^ T cell subset in the FRT, are particularly susceptible to HIV-1 infection [43,49–52]. The tissue sections were labeled with immunofluorescent antibodies against CD3, CCR6, and Gag and counterstained with DAPI (**Fig 4**) as per the established approach for identifying infected Th17 cells [53–57]. In addition to infected Th17 cells (**Fig 4E**), other cells were also infected, including cells expressing CCR6 but not CD3, which could be immature dendritic cells (DCs) (**Fig 4**). Immature DCs express CCR6 and are susceptible to HIV-1 infection, and our lab has demonstrated this in our NHP studies [14,44]. Notably, the infected cells observed here were very few, making our interpretation of the differential susceptibility of endo and ectocervix to HIV-1 infection inconclusive. Therefore, we could not proceed with this work and remained exploratory. However, we demonstrated that T/F HIV-1 variants can infect diverse cell phenotypes, ranging from T cells, non-T cells, and immature DCs, similar to data obtained from our NHP studies [14,42,58]. NHP models are valuable for studying HIV/SIV infection, providing insights into viral kinetics and immune responses that are challenging to obtain in humans [59,60]. These models facilitate the investigation of early infection events, viral kinetics, and the distribution of immune cells across various tissues [61,62]. NHPs show similarities to humans in Mucosal-Associated Invariant T (MAIT) cell frequencies and NK cell biology, both of which are crucial for understanding HIV pathogenesis [63,64]. MAIT cells, important for mucosal defense, are depleted during HIV/SIV infection in both humans and NHPs [63]. The distribution and function of NK cells vary across tissues and primate species, both at steady state and during SIV infection [62,64]. However, there are pharmacological differences between NHPs and humans, as recently demonstrated in *ex vivo* tissue explant studies [65]. Furthermore, NHPs generally exhibit lower acute viremia and less disruption of gut CD4^+^ T-cell homeostasis during SHIV infection compared to SIV infection [61]. These differences underscore the need for careful interpretation of results from NHP models when translating to human applications. This is why our *ex vivo* human explant work is essential for addressing these differences. Thus, our findings should be confirmed through studies employing hypothesis-driven testing to comprehensively phenotype the infected cells and determine whether these T/F HIV-1 variants infect specific immune cells, which may help explain the differential susceptibility of endocervical and ectocervical tissues to HIV-1 infection that we observed.

### Nevirapine partially inhibited HIV-1 replication in cervical tissues but did not completely stop it

Antiretroviral therapy (ART) is a treatment that is used for HIV-1 management [66–68]. While effective, ART fails to stop HIV-1 infection and replication in our research entirely. In our study, we used NVP at a higher dose than what is commonly given to HIV-1 infected patients so as to have a maximum inhibition of HIV replication. This was done to distinguish the *de novo* production of HIV-1 DNA from the viral inoculum, as most T/F HIV-1 variants generally do not replicate efficiently to high levels across different tissue types and cell populations [69–72]. We selected NVP because it rapidly dissolves and penetrates tissues and is effective against both cell-associated and free HIV-1 virions [73–75].

**Fig 5:**
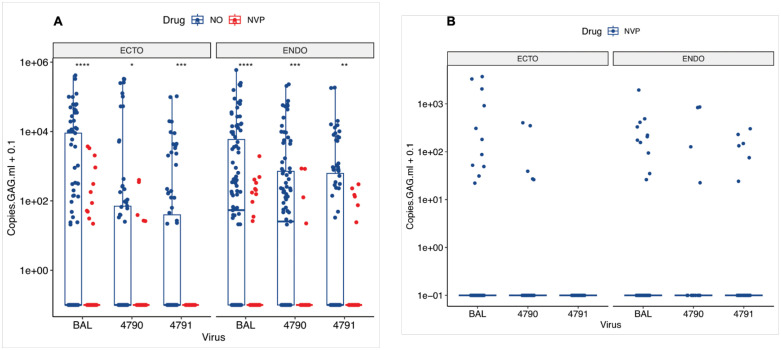
Nevirapine suppressed but did not block HIV-1 replication in cervical tissues. Donor-matched endocervical and ectocervical explants were infected either with HIV-1_BaL_ (Endo; N = 202; Ecto; N = 181), HIV-1_4790_ (Endo; N = 123; Ecto; N = 110)_,_ or HIV-1_4791_ (Endo; N = 128; Ecto; N = 122). NVP-treated cultures were performed alongside them and cultured for 12 days. **A**) Comparison of HIV-1 copies in tissues with and without NVP; **B**) Comparison of the rate of HIV-1 inhibition by NVP within tissues that had replication. The boxplot presents the median and quartile ranges and whiskers extending to 1.5 times the max and min-quartile values of the HIV-1 DNA copies at the end of the culture. A single point represents each sample analyzed. Asterisks denote adjusted p-values: * < 0.05,** < 0.01,*** < 0.001, **** < 0.0001.

Endocervical and ectocervical tissues treated with NVP had significantly lower HIV-1 DNA copies than those without (**Fig 5A**). When we compared the tissue type and the virus, we observed that tissues infected with HIVBaL without NVP were significantly higher than those with NVP in both the endocervix and ectocervix (p < 0.0001; **Fig 5A**). The same was seen for HIV_4790_ infected endocervix (p < 0.01) and ectocervix (p < 0.05). Interestingly, in HIV_4791_ infected ectocervical explants, NVP completely inhibited HIV-1 replication (p < 0.0001), but was not in matched endocervical explants, where we observed some replication, although significantly lower than in HIV-1 infected explants without NVP (p < 0.01; **Fig 5A**). The factors contributing to this difference in NVP activity against the virus in these two tissues require further investigation. When we compared the activity of NVP in the endocervix and ectocervix tissues across the three viruses, we found no significant differences in HIV-1 copies (**Fig 5B**). In summary, we observed that NVP-treated endocervical and ectocervical tissues infected with HIV_BaL_ were more resistant to NVP compared to those infected with T/F HIV-1 variants. This suggests that T/F HIV-1 variants may be more sensitive to NVP than HIV_BaL_, but additional research would be necessary to confirm our results.

The use of NVP in our experiments has immediate practical implications. We could differentiate the newly produced HIV-1 from the original viral inoculum. Although NVP did not wholly block the replication of HIV-1 in some tissues, it suppressed replication in most tissues, similar to its effects *in vivo*, where it does not entirely block HIV-1 replication but reduces it to undetectable levels in people with HIV on ART [76]. In summary, our findings on the use of NVP as a control for HIV-1 infection studies have practical implications for identifying and quantifying the new HIV production, which is important when testing isolated T/F HIV-1 variants that typically do not replicate efficiently in tissues.

## Discussion

HIV remains a significant public health threat, and understanding the earliest events during transmission is crucial for developing effective therapies and vaccines. However, models of HIV-1 transmission based on rhesus macaques may not fully translate to humans [12,77–81]. Due to this, human cervical tissue explants have been used as an alternative model to study the mucosal transmission of HIV-1 [19,82–84]. In this study, we investigated the susceptibility of ecto- and endocervical tissues to HIV-1 infection using an *ex vivo* model platform. Our results showed that most of the cervical tissues tested were productively infected with T/F HIV-1 variants and reference HIV-1_BaL_ strain, indicating that the cervical tissues were highly susceptible to HIV-1 infection. Our research suggests that the endocervix and ectocervix exhibit differential susceptibility to T/F HIV-1 variants and HIV-1_BaL_. The endocervix shows higher levels of HIV-1 infection and replication compared to the ectocervix, contrary to previous studies [85,86]. Lastly, we evaluated the use of nevirapine as a control for differentiating the *de novo* production of HIV-1 from the original viral inoculum. We found it important as it significantly inhibited HIV-1 replication but did not entirely block it.

First, we showed that T/F HIV-1 variants can infect most cervical tissue *ex vivo,* and infected cervical tissues sustained HIV-1 replication in culture over time. This is important in assessing the viral dynamics in tissues infected by T/F HIV-1 variants that typically do not replicate efficiently or are slow replicators. Due to this, we used a reference HIV-1_BaL_ and NVP to determine the replicative capacity of the T/F HIV-1 variants and also detect and quantify *de novo* production of HIV-1. Due to the inefficiency of replicating T/F HIV-1 variants in tissues, we deliberately used a higher dosage of NVP in our experiments than the one used in the treatment of patients with HIV to achieve complete inhibition of viral replication. We observed significant inhibition of viral replication in most NVP-treated tissues compared to untreated cultures; however, some HIV-1 replication was observed in certain tissues. Specifically, we observed some replication in NVP-treated endocervical tissues infected with HIV_4791_, unlike donor-matched ectocervical tissues, which had complete inhibition. One plausible reason for this could be the presence of endocervical mucus, which may trap the drug, reducing its bioavailability. Other potential factors could be that the viral strains tested here are resistant to the NVP; however, this remains to be investigated. While NVP suppresses viral replication, immune activation persists in treated tissues, as evidenced by elevated cytokine levels [87], leading to viral replication in some NVP-treated tissues and underscoring the efficacy of antiretroviral therapies. Additionally, off-target effects from escalating doses of NVP might influence viral replication. Studies have shown that NVP can induce mitochondrial depolarization and apoptosis in lymphocytes, potentially reducing viral replication [88]. Furthermore, NVP also activates immune responses, including MHC Class I activation and cytokine upregulation [87,89], potentially increasing viral replication. This should be considered in HIV infection studies and other interventions such as microbicides. Although research on T/F HIV-1 variants has revealed important insights into viral transmission and replication, the results have been inconsistent. While some studies suggest successful infection across different anatomical sites, including anogenital tissues [90], colorectal tissues [91], rectosigmoid and tonsillar tissues [92], and human penile and cervical mucosa tissues [82], others indicate aborted infection [15]. These inconsistencies may be attributed to the differences in the *ex vivo* culture model platforms employed by different research groups. The T/F HIV-1 variants might also be a factor, as some studies have reported that the HIV-1 clades may influence viral replication, with HIV-1 clade A and recombinant A/G replicating more efficiently than clade C and clade B T/F HIV-1 variants [93]. The T/F HIV-1 variants used in this study are clade C viruses. Thus, demonstrating the infectivity of these variants in cervical tissues will contribute to the pool of knowledge about the replication patterns of T/F HIV-1 variants in cervical tissues. We believe we are the first group to characterize these variants in cervical tissues *ex vivo*, thus contributing to a better understanding of HIV transmission and pathogenesis in the cervical mucosa. These T/F HIV-1 variants are biologically relevant for studying HIV transmission and developing prevention strategies compared to control viruses such as HIV-1_BaL_ [94]. HIV-1_BaL_ is commonly used in *ex vivo* HIV infection studies, as it replicates more efficiently in cervical and vaginal tissues than other strains, making it suitable for *ex vivo* mucosal transmission models [85,95–98]. Therefore, we selected this strain in our studies as the reference point to compare the infection and replication efficiency of the T/F strains in cervical tissue tissues. Our results align with previous studies, in which we observed higher replication levels of HIV-1_BaL_ in endocervical and ectocervical tissues; however, these levels varied across tissues and donors (**Fig 2**). Our main goal was not to compare the infection and replication levels between HIV-1_BaL_ and T/Fs, but to use HIV-1_BaL_ as a reference to compare the susceptibility of the endocervical and ectocervical tissues to T/Fs strains. Both HIV-1 variants and HIV-1_BaL_ have demonstrated similar efficiency in utilizing CD4 and CCR5 [99], exhibit Tier 2 neutralization sensitivity, and are more sensitive to some broadly neutralizing antibodies [100,101]. Furthermore, Clade C T/F SHIVs have been developed for vaccine research in NHP, mirroring the characteristics of natural T/F viruses [102]. Due to these characteristics, T/F HIV-1 variants are crucial for developing effective HIV-1 prevention strategies and vaccines, and our findings will contribute to this undertaking.

Our study focused on Clade C T/F HIV variants, the predominant and circulating variants in Sub-Saharan Africa. These variants were isolated from the acute HIV cohort from sub-Saharan Africa [26] and were shown to replicate efficiently in the PBMC *in vitro* [103]. Thus, our *ex vivo* data complement these *in vitro* results and validate our *ex vivo* culture model platform for HIV infection studies in the cervical mucosa and other anatomical sites. *Ex vivo* studies at anatomical sites, a primary portal of HIV transmission, are crucial. Studies have shown that T/F HIV-1 variants can replicate efficiently in peripheral blood *in vitro,* but poorly in tissues, or do not replicate at all [82]. This can be attributed to many factors, including the type of cells in each compartment, their activation status, and antiviral mediators, among others.

*Ex vivo* studies using human cervicovaginal, rectal, and tonsillar tissues have offered valuable insights into HIV-1 transmission and pathogenesis. These models allow for the investigation of early infection events and the evaluation of prevention strategies [85,104]. Our research employed a non-polarized approach to challenge ectocervical and endocervical tissues with HIV-1. We cultured the exposed tissues at the liquid-air interface, using gelatin sponges as a support, which maximized exposure to air oxygen while providing access to culture medium nutrients through the sponge capillaries, thereby delaying their natural decay. In contrast to prior studies that have shown ectocervical tissues support higher levels of HIV-1 replication [85,86,105], our analysis indicates that endocervical tissues may be more susceptible to HIV-1 than ectocervical tissues. This is consistent with other studies that have shown that the endocervix could be more vulnerable to HIV-1 infection [45,106]. Our results contrast with prior studies that reported ectocervical tissues as more susceptible to HIV/SIV infection than endocervical tissues [11–13]. This discrepancy may be attributed to several potential factors, including the viral strains used; most of those studies employed lab-adapted BaL strains, whereas we utilized primary isolates from clade C T/F, which are predominant in the sub-Saharan region where our study was conducted. Additionally, the experimental setups differ across studies: we used non-polarized explants without exogenous PHA activation, unlike the methods employed by other studies [107–111], which may explain the variability of results between previous studies and our findings. All these variables—viral strains, tissue handling and preparation, and experimental conditions—may significantly impact the outcomes of studies and explain the differences in results obtained by various researchers. In our study, we used an identical batch of viral stocks to maintain uniformity in infection throughout the study, and we employed identical tissue preparation across experimental conditions whenever possible to ensure comparable results. Nonetheless, these *ex vivo* models provide valuable insights into early HIV-1 infection events and serve as platforms for evaluating potential HIV prevention strategies.

Previous studies may have illustrated that the endocervix is more susceptible to HIV infection, with a plausible explanation being that the presence of Th17 cells—which we and others have demonstrated in NHP and *ex vivo* models to be preferentially susceptible to HIV—might be one of the factors influencing this [14,112,113]. Th17 cells are a subset of CD4^+^ T cells characterized by their production of IL-17 cytokines, particularly IL-17A and IL-17F [114–117]. They play a crucial role in host defense against extracellular bacteria and fungi [118,119]. Primarily located in mucosal tissues, Th17 cells contribute to maintaining mucosal barrier integrity [114]. In the current study, we did not investigate the infectivity of these cells due to limitations in the tissues; instead, we prioritized the analysis of CD3^+^ T cells for their infectivity. The goal of this study was to determine the susceptibility of the ectocervical and endocervical tissues to various viral strains, regardless of the phenotype of the infected cells. However, based on exploratory experiments involving immunofluorescent staining of tissues to investigate the presence of CD3^+^ T cells, we observed a widespread distribution of infected CD3^+^ T cells in the epithelium, lamina propria, and stroma. At this stage, we did not quantify or phenotype the subpopulation of infected CD3^+^ T cells as it was beyond the scope of the study. Whether their abundance in the ectocervix and endocervix, along with the phenotype of infected cells, contributes to the differential susceptibility requires further investigation.

The variability of tissue infectivity in donors may stem from several factors, including the menstrual cycle. The phase of the menstrual cycle can influence tissue susceptibility to HIV-1 in women, with the luteal/secretory phase associated with an increased risk [120,121]. Studies in macaques and human tissue explants demonstrate a higher susceptibility to SHIV/HIV infection during the luteal/secretory phase [122,123]. This heightened vulnerability may result from the hormonal regulation of HIV receptors and coreceptors on epithelial cells [124], as well as enhanced immune activation and epithelial barrier remodeling [125]. Progesterone levels correlate with higher frequencies of HIV-targeting CD4 + α4β7 + T cells and stronger pro-inflammatory responses [121]. Throughout the late luteal phase, cyclical fluctuations in CCR5^+^ memory CD4^+^ T cell infiltration into the vaginal lumen occur [126]. Additionally, transcriptome analysis indicates menstrual cycle-associated changes in both the endocervix and ectocervix that may influence HIV susceptibility [120]. Moreover, studies suggest that hormonal changes during menopause significantly affect mucosal immune responses and HIV-1 susceptibility in the FRT. Postmenopausal women show increased HIV-1 infectivity, a higher frequency of target cells, decreased tight junction proteins, altered immune cell composition, and increased CCR5 expression on cervical CD4^+^ T cells, alongside heightened immune activation and epithelial thinning, likely due to estradiol deficiency and progestin exposure [43,127–129]. The primary mechanisms behind this are reduced barrier integrity, increased inflammation, superficial HIV target cells, glucocorticoid receptor-mediated upregulation, and immune cell redistribution [43,127–129]. Conversely, premenopausal women exhibit lower HIV infection rates and a low frequency of CCR5-positive cells due to higher estradiol levels, a robust epithelial barrier, and reduced inflammation [130]. Older women exhibit changes in the vaginal microbiome, heightened inflammation, and a decrease in Lactobacillus species, which may potentially increase risk [131]. Collectively, these changes, alongside immunosenescence, contribute to increased susceptibility to sexually transmitted infections such as HIV in aging women [132]. Postmenopausal women show increased susceptibility to HIV and other infections due to a thinned vaginal epithelium, altered immune factors, and changes in the vaginal microbiome. These alterations are primarily attributed to estrogen deficiency. Estrogen treatment can reverse these effects, restoring mucosal conditions to premenopausal levels. Injectable contraceptives like depot medroxyprogesterone acetate (DMPA) have demonstrated conflicting results regarding HIV susceptibility. Estrogen may offer protective effects against HIV-1 infection, while progesterone and synthetic progestins like medroxyprogesterone acetate may heighten susceptibility [133,134]. While DMPA reduces bacterial vaginosis, it increases immune cell numbers and activation markers in vaginal tissues [135]. The vaginal microbiome plays a crucial role in mucosal immunity, with estrogen favoring a Lactobacillus-dominated environment [136,137]. A higher abundance of immune factors important for HIV susceptibility, including antiproteases and antimicrobial factors, have also been attributed to increasing susceptibility [138]. Unfortunately, these factors were beyond the scope of this study, and further research is needed to fully understand how they may contribute to the increased susceptibility of the endocervix to HIV-1 infection.

While our study provides valuable insights into the susceptibility of cervical tissue to HIV infection, it is important to acknowledge its limitations. Firstly, the sample size was limited, and we only utilized two clade C HIV-1 T/Fs variants; therefore, our results may not be generalized to other HIV-1 T/Fs or clades. Secondly, we could only measure HIV-1 replication at a single time due to limited tissue acquisition. In doing so, we could not account for the fact that some T/F HIV-1 variants are fast replicators while others are slow replicators, as more samples would be needed for time-course experiments [103]. Also, we measured HIV-1 replication using RT-qPCR, which fails to distinguish HIV-1 DNA from actively replicating cells and that released by apoptotic cells; therefore, the results should be interpreted cautiously. Another limitation of our study is that women undergoing hysterectomy tend to be older, premenopausal, or postmenopausal and generally have associated conditions that are responsible for the hysterectomy, such as fibroids, cancer, etc. [139–141]. Additionally, we did not collect information on the menstrual cycle phase and contraceptive use. All these factors may affect the immune cell composition in the genital tract, thus impacting the susceptibility of cervical tissue to HIV-1 infection [142–145]. Future investigations should, therefore, collect additional clinical information such as hormonal status, sexual activity, history of sexually transmitted infections, tobacco use, and use of intrauterine devices of the participants to understand better the factors that may influence the susceptibility of cervical tissue to HIV-1 infection and replication. Finally, explants do not accurately mimic the natural infection and complex events happening during *in vivo* transmission when other variables, such as semen, vaginal flora, and pH, come into play. However, despite these limitations of an *ex vivo* model, our findings provide strong evidence of the value of human cervical tissue explants in HIV-1 infection studies and suggest that the endocervix may be more vulnerable to HIV infection than the ectocervix.

In summary, we have studied the replication dynamics of Clade C T/F HIV-1 variants in cervical tissues, and to the best of our knowledge, we are the first group to do so in a sub-Saharan cohort from which the T/F variants were isolated. Our findings demonstrate that the upper and lower cervical tissues are not equally susceptible to HIV-1 infection *ex vivo*. We observed more infection and replication of HIV-1 in the endocervix compared to the ectocervix. Therefore, further research is necessary to understand the mechanisms underlying this differential susceptibility between these two distinct regions of the cervix to HIV-1. Overall, our results will contribute to the understanding of HIV pathogenesis in cervical mucosa, which may help in designing more effective and targeted prevention strategies against HIV-1.

## Supporting information

S1 Fig GAPDH levels were similar across samples. Donor-matched cervical tissue blocks were either infected with HIV-1, infected with HIV-1, and treated with NVP, or uninfected (control) and cultured for 12 days. The boxplot presents the median and quartile ranges and whiskers extending to 1.5 times the max and min-quartile values of the numbers of GAPDH copies on day 12. A single point represents each sample analyzed. There was no significant difference between the groups. The Kruskal-Wallis test was used for statistical analysis. NVP; nevirapine, NO denotes cultures without NVP, NEG; control.
(PDF)

S1 Table HIV-1 replication levels in cervical tissues. (PDF)
