# Supplementary figures and images for: Endocervix exhibits greater susceptibility to HIV-1 infection compared to ectocervix following ex vivo exposure to Transmitted/Founder HIV-1 variants
Source: PLoS One. 2025 Nov 5;20(11):e0334510. doi: 10.1371/journal.pone.0334510 (PMC12588514; doi:10.1371/journal.pone.0334510)

S1 Fig

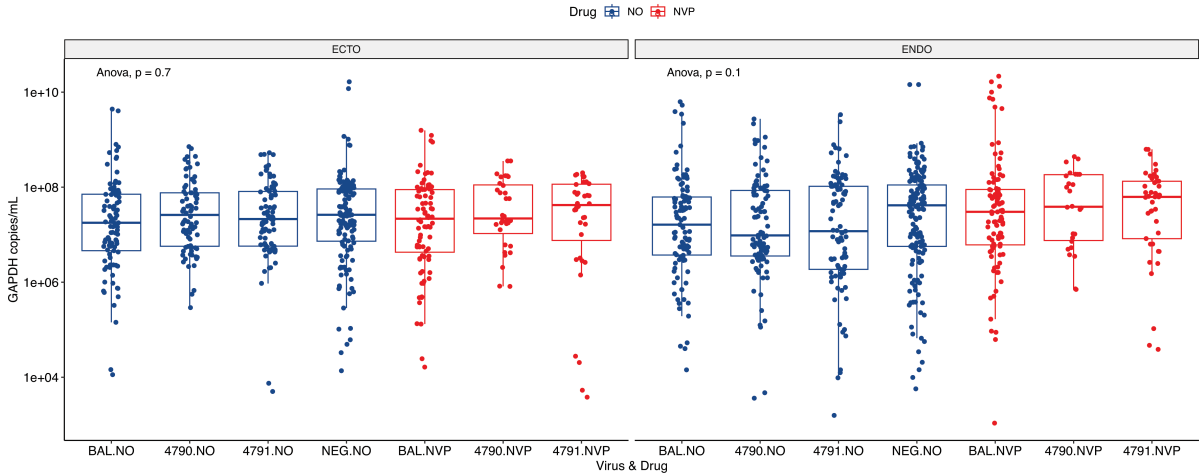

Supplement: S1 Fig — Donor-matched cervical tissue blocks were either infected with HIV-1, infected with HIV-1, and treated with NVP, or uninfected (control) and cultured for 12 days. The boxplot presents the median and quartile ranges and whiskers extending to 1.5 times the max and min-quartile values of the numbers of GAPDH copies on day 12. A single point represents each sample analyzed. There was no significant difference between the groups. The Kruskal-Wallis test was used for statistical analysis. NVP; nevirapine, NO denotes cultures without NVP, NEG; control. (PDF) [file pone.0334510.s001.pdf]
