# Supplementary material for: Endocervix exhibits greater susceptibility to HIV-1 infection compared to ectocervix following ex vivo exposure to Transmitted/Founder HIV-1 variants
Source: PLoS One. 2025 Nov 5;20(11):e0334510. doi: 10.1371/journal.pone.0334510 (PMC12588514; doi:10.1371/journal.pone.0334510)
Supplement: S1 Table — (PDF) [file pone.0334510.s002.pdf]

S1 Table

	ECTO			ENDO		
**Characteristic**	**BAL**	**4790**	**4791**	**BAL**	**4790**	**4791**
Copies.GAG.ml						
N	41	25	23	56	48	31
Median (IQR)	11,438 (334, 43,879)	259 (90, 53,497)	2,303 (168, 6,716)	3,709 (251, 19,491)	776 (187, 11,004)	943 (438, 8,483)
Range	34, 417,025	33, 329,657	27, 103,697	32, 595,119	29, 229,490	33, 184,887
